# Supplementary material for: Educational Preparation and Course Approach of Undergraduate Sports Nutrition instructors in Large U.S. Institutions
Source: Sports (Basel). 2023 Sep 5;11(9):176. doi: 10.3390/sports11090176 (PMC10536551; doi:10.3390/sports11090176)
Supplement: Supplementary file 1 [file sports-11-00176-s001.zip › sports-2564794-supplementary survey.pdf]

Sports Nutrition Course Evaluation

Start of Block: Course Demographics

Q1 Please provide your name and institution. These will be de-identified once data has been collected

____

Q2 Please upload a copy of your class syllabus (optional)

Q3 Which department is your course in?

____

Q4 What is the course number?

____

Q5 Which classes, if any, are required as pre-requisites to taking your sports nutrition class? (Select all that apply)

- ▢ Basic nutrition (1)
- ▢ Nutrient metabolism (2)
- ▢ Exercise physiology (3)
- ▢ Biochemistry (4)
- ▢ Human physiology (5)
- ▢ Other, please specify (6) ____

Q6 How many credits is your course?

▼ 1 credit (1) ... More than 4 credits (5)

Q7 How many weeks is this course taught in a normal semester?

▼ Less than 12 weeks (1) ... More than 15 weeks (6)

Q8 How much experience do you have teaching this course?

- ◯ Preparing for 1st semester (1)
- ◯ 1-2 semesters (2)
- ◯ 3-4 semesters (3)
- ◯ 5-6 semesters (4)
- ◯ More than 6 semesters (5)

Q9 Is a textbook required?

- ◯ Yes (1)
- ◯ No (2)

Skip To: Q13 If Is a textbook required? = No

Q10 Rate on a scale of 1-10 how important the textbook is, with 1 being not very important and 10 being extremely important

	1	2	3	4	5	6	6	7	8	9	10
Textbook use ()											

Display This Question:

If Is a textbook required? = Yes

Q11 What textbook do you use for your course?

▼ Benardot, Dan. ACSM's Nutrition for Exercise Science. Philidelphia, PA: Wolters Kluwer, 2019. (1) ... My textbook is not listed (11)

Skip To: Q12 If What textbook do you use for your course? = My textbook is not listed

Q12 What textbook do you use?

____

Q13 Do you have supplemental readings?

- ○ Yes (1)
- ○ No (2)

Skip To: Q15 If Do you have supplemental readings? = No

Q14 Rate on a scale of 1-10 how important your supplemental readings are, with 1 being not very important and 10 being extremely important

	1	2	3	4	5	6	6	7	8	9	10
Supplemental readings ()											

Page Break

Q15 In what field did you receive your Bachelor's degree?

____

Q16 Did you receive a Master's degree?

- Yes (1)
- No (2)

Skip To: Q18 If Did you receive a Master's degree? = No

Q17 In what field did you receive your Master's degree?

____

Q18 Do you have a doctoral degree?

- Yes (1)
- No (2)

Skip To: Q20 If Do you have a doctoral degree? = No

Q19 In what field did you receive your doctoral degree?

____

Q20 What additional credentials do you have, if any? (Select all that apply)

- ☐ Registered Dietitian Nutritionist (RDN) (1)
- ☐ Certified Sports Dietitian (CSSD) (2)
- ☐ Certified Nutrition Specialist (CNS) (3)
- ☐ ACSM Certified Personal Trainer (ACSM-CPT) (4)
- ☐ ACSM Certified Exercise Physiologist (ACSM-EP) (5)
- ☐ ACSM Certified Group Exercise Instructor (ACSM-GEI) (6)
- ☐ Certified Inclusion Fitness Trainer (CIFT) (7)
- ☐ Physical Activity in Public Health Specialist (PAPHS) (8)
- ☐ Other, please specify (9) ____

End of Block: Course Demographics

Start of Block: Evaluation of Course Content

Q21 About how much time do you typically spend on each topic related to **carbohydrates** in a given semester?

	Not taught (1)	1-5 minutes (2)	6-10 minutes (3)	11-15 minutes (4)	16-20 minutes (5)	More than 20 minutes (6)
Digestion and absorption (1)	o	o	o	o	o	o
Utilization (2)	o	o	o	o	o	o
Storage (3)	o	o	o	o	o	o
Metabolic pathways (4)	o	o	o	o	o	o
Functions (5)	o	o	o	o	o	o
Estimating needs (6)	o	o	o	o	o	o
Structures (7)	o	o	o	o	o	o
Dietary sources (8)	o	o	o	o	o	o
Sample diets (9)	o	o	o	o	o	o
Timing of recommendations (10)	o	o	o	o	o	o
Carbohydrate loading (11)	o	o	o	o	o	o

Q22 Rate from 1-10 based on how deep you go into each of these topics related to **carbohydrates**, with 1 being very little depth and 10 being extremely deep.

Not Taught

	1	2	3	4	5	6	7	8	9	10
Digestion and absorption ()										
Utilization ()										
Storage ()										
Metabolic pathways ()										
Functions ()										
Estimating needs ()										
Structures ()										
Dietary sources ()										
Sample diets ()										
Timing of recommendations ()										
Carbohydrate loading ()										

Q23 Rate from 1-10 based on how important you think each of these topics are related to **carbohydrates** with 1 being not important and 10 being extremely important.

Not Taught

	1	2	3	4	5	6	7	8	9	10
Digestion and absorption ()										
Utilization ()										
Storage ()										
Metabolic pathways ()										
Functions ()										
Estimating needs ()										
Structures ()										
Dietary sources ()										
Sample diets ()										
Timing of recommendations ()										
Carbohydrate loading ()										

Page Break

Q24 About how much time do you typically spend on each topic related to **fats** in a given semester?

	Not taught (1)	1-5 minutes (2)	6-10 minutes (3)	11-15 minutes (4)	16-20 minutes (5)	More than 20 minutes (6)
Digestion and absorption (1)	o	o	o	o	o	o
Utilization (2)	o	o	o	o	o	o
Storage (3)	o	o	o	o	o	o
Metabolic pathways (4)	o	o	o	o	o	o
Functions (5)	o	o	o	o	o	o
Estimating needs (6)	o	o	o	o	o	o
Structures (7)	o	o	o	o	o	o
Dietary sources (8)	o	o	o	o	o	o
Sample diets (9)	o	o	o	o	o	o
Timing of recommendations (10)	o	o	o	o	o	o
Fat loading (11)	o	o	o	o	o	o

Q25 Rate from 1-10 based on how deep you go into each of these topics related to **fats**, with 1 being very little depth and 10 being extremely deep.

	Not Taught 1 2 3 4 5 6 7 8 9 10
Digestion and absorption ()	
Utilization ()	
Storage ()	
Metabolic pathways ()	
Functions ()	
Estimating needs ()	
Structures ()	
Dietary soures ()	
Sample diets ()	
Timing of recommendations ()	
Fat loading ()	

Q26 Rate from 1-10 based on how important you think each of these topics are related to **fats** with 1 being not important and 10 being extremely important.

	Not Taught									
	1	2	3	4	5	6	7	8	9	10
Digestion and absorptions ()										
Utilization ()										
Storage ()										
Metabolic pathways ()										
Functions ()										
Estimating needs ()										
Structures ()										
Dietary sources ()										
Sample diets ()										
Timing of recommendations ()										
Fat loading ()										

Page Break

Q27 About how much time do you typically spend on each topic related to **proteins** in a given semester?

	Not taught (1)	1-5 minutes (2)	6-10 minutes (3)	11-15 minutes (4)	16-20 minutes (5)	More than 20 minutes (6)
Digestion and absorption (1)	o	o	o	o	o	o
Utilization (2)	o	o	o	o	o	o
Storage (3)	o	o	o	o	o	o
Metabolic pathways (4)	o	o	o	o	o	o
Functions (5)	o	o	o	o	o	o
Estimating needs (6)	o	o	o	o	o	o
Structures (7)	o	o	o	o	o	o
Dietary sources (8)	o	o	o	o	o	o
Sample diets (9)	o	o	o	o	o	o
Timing of recommendations (10)	o	o	o	o	o	o

Q28 Rate from 1-10 based on how deep you go into each of these topics related to **proteins**, with 1 being very little depth and 10 being extremely deep.

	Not Taught									
	1	2	3	4	5	6	7	8	9	10
Digestion and absorption ()										
Utilization ()										
Storage ()										
Metabolic pathways ()										
Functions ()										
Estimating needs ()										
Structures ()										
Dietary sources ()										
Sample diets ()										
Timing of recommendations ()										

Q29 Rate from 1-10 based on how important you think each of these topics are related to **proteins** with 1 being not important and 10 being extremely important.

	Not Taught									
	1	2	3	4	5	6	7	8	9	10
Digestion and absorption ()										
Utilization ()										
Storage ()										
Metabolic pathways ()										
Functions ()										
Estimating needs ()										
Structures ()										
Dietary sources ()										
Sample diets ()										
Timing of recommendations ()										

Page Break

Q30 About how much time do you typically spend on each topic related to **body composition and weight management** in a given semester?

	Not taught (1)	1-5 minutes (2)	6-10 minutes (3)	11-15 minutes (4)	16-20 minutes (5)	More than 20 minutes (6)
Determination of healthy body weights (1)	o	o	o	o	o	o
Appropriate weight loss/gain (2)	o	o	o	o	o	o
Percent body fat in males vs females (3)	o	o	o	o	o	o
Sports specific percent body fat (4)	o	o	o	o	o	o
Tightly controlled prescriptive diets vs more liberal diet approaches (5)	o	o	o	o	o	o
Body composition goals for athletes (6)	o	o	o	o	o	o
Methods of determining body composition (7)	o	o	o	o	o	o
Methods of determining healthy body weight (BMI, composition, performance) (8)	o	o	o	o	o	o
Determining energy needs	o	o	o	o	o	o

(9)						
Changes in body composition due to age, gender, menopause, type of activity, genetic predisposition, etc. (10)	○	○	○	○	○	○
Weight cutting practices (11)	○	○	○	○	○	○

Q31 Rate from 1-10 based on how deep you go into each of these topics related to **body composition and weight management**, with 1 being very little depth and 10 being extremely deep.

	Not Taught<br>1 2 3 4 5 6 7 8 9 10
Determination of healthy body weights ()	
Appropriate weight loss/gain ()	
Percent body fat in males vs females ()	
Sports specific percent body fat ()	
Tightly controlled prescriptive diets vs more liberal diet approaches ()	
Body composition goals for athletes ()	
Methods of determining body composition ()	
Methods of determining healthy body weight (BMI, composition, performance) ()	
Determining energy needs ()	
Changes in body composition due to age, gender, menopause, type of activity, genetic predisposition, etc. ()	
Weight cutting practices ()	

Q32 Rate from 1-10 based on how important you think each of these topics are related to **body composition and weight management** with 1 being not important and 10 being extremely important.

	Not Taught<br>1 2 3 4 5 6 7 8 9 10
Determination of healthy body weights ()	
Appropriate weight loss/gain ()	
Percent body fat in males vs females ()	
Sports specific percent body fat ()	
Tightly controlled prescriptive diets vs more liberal diet approaches ()	
Body composition goals for athletes ()	
Methods of determining body composition ()	
Methods of determining healthy body weight (BMI, composition, performance) ()	
Determining energy needs ()	
Changes in body composition due to age, gender, menopause, type of activity, genetic predisposition, etc. ()	
Weight cutting practices ()	

Page Break

Q33 About how much time do you typically spend on each topic related to **fluids** in a given semester?

	Not taught (1)	1-5 minutes (2)	6-10 minutes (3)	11-15 minutes (4)	16-20 minutes (5)	More than 20 minutes (6)
Functions (1)	o	o	o	o	o	o
Measuring water balance (2)	o	o	o	o	o	o
Over and under hydration, including fluid related problems (3)	o	o	o	o	o	o
Calculating fluid loss (4)	o	o	o	o	o	o
Timing (before, during, after exercise) (5)	o	o	o	o	o	o
Assessing fluid needs (6)	o	o	o	o	o	o
Environmental factors (7)	o	o	o	o	o	o
Absorption (8)	o	o	o	o	o	o

Q34 Rate from 1-10 based on how deep you go into each of these topics related to **fluids**, with 1 being very little depth and 10 being extremely deep.

	Not Taught<br>1 2 3 4 5 6 7 8 9 10
Functions ()	
Measuring water balance ()	
Over and under hydration, including fluid related problems ()	
Calculating fluid loss ()	
Timing (before, during, and after exercise) ()	
Assessing fluid needs ()	
Environmental factors ()	
Absorption ()	

Q35 Rate from 1-10 based on how important you think each of these topics are related to **fluids** with 1 being not important and 10 being extremely important.

	Not Taught
	1 2 3 4 5 6 7 8 9 10
Functions ()	
Measuring water balance ()	
Over and under hydration, including fluid related problems ()	
Calculating fluid loss ()	
Timing (before, during, and after exercise) ()	
Assessing fluid needs ()	
Environmental factors ()	
Absorption ()	

Page Break

Q36 About how much time do you typically spend on each topic related to **eating disorders** in a given semester?

	Not taught (1)	1-5 minutes (2)	6-10 minutes (3)	11-15 minutes (4)	16-20 minutes (5)	More than 20 minutes (6)
Types of eating disorders (1)	○	○	○	○	○	○
Orthorexia (2)	○	○	○	○	○	○
Female athlete triad (3)	○	○	○	○	○	○
Disordered eating (4)	○	○	○	○	○	○
Signs and symptoms (5)	○	○	○	○	○	○
Causes (6)	○	○	○	○	○	○
Treatments (7)	○	○	○	○	○	○
Hormone adaptations (8)	○	○	○	○	○	○

Q37 Rate from 1-10 based on how deep you go into each of these topics related to **eating disorders**, with 1 being very little depth and 10 being extremely deep.

	Not Taught
	1 2 3 4 5 6 7 8 9 10
Types of eating disorders ()	
Orthorexia ()	
Female athlete triad ()	
Disordered eating ()	
Signs and symptoms ()	
Causes ()	
Treatments ()	
Hormone adaptations ()	

Q38 Rate from 1-10 based on how important you think each of these topics are related to **eating disorders** with 1 being not important and 10 being extremely important.

Not Taught

	1	2	3	4	5	6	7	8	9	10
Types of eating disorders ()										
Orthorexia ()										
Female athlete triad ()										
Disordered eating ()										
Signs and symptoms ()										
Causes ()										
Treatments ()										
Hormone adaptations ()										

Page Break

Q39 About how much time do you typically spend in class on each of these **vitamins and minerals** in a given semester?

	Not Taught (1)	1-5 minutes (2)	6-10 minutes (3)	11-15 minutes (4)	16-20 minutes (5)	More than 20 minutes (6)
Vitamin A (1)	○	○	○	○	○	○
Vitamin D (2)	○	○	○	○	○	○
Vitamin E (3)	○	○	○	○	○	○
Vitamin K (4)	○	○	○	○	○	○
Vitamin B12 (5)	○	○	○	○	○	○
Folic acid (6)	○	○	○	○	○	○
Other B vitamins (7)	○	○	○	○	○	○
Vitamin C (8)	○	○	○	○	○	○
Calcium (9)	○	○	○	○	○	○
Sodium (10)	○	○	○	○	○	○
Potassium (11)	○	○	○	○	○	○
Iron (12)	○	○	○	○	○	○
Other minerals not included in this list (13)	○	○	○	○	○	○

Q40 About how much time do you typically spend in class on each of these **ergogenic aids** in a given semester?

	Not Taught (1)	1-5 minutes (2)	6-10 minutes (3)	11-15 minutes (4)	16-20 minutes (5)	More than 20 minutes (6)
Caffeine (1)	o	o	o	o	o	o
Sodium bicarbonate (2)	o	o	o	o	o	o
Creatine (3)	o	o	o	o	o	o
Nitrate (4)	o	o	o	o	o	o
Beta-alanine (5)	o	o	o	o	o	o
Leucine (6)	o	o	o	o	o	o
Carnitine (7)	o	o	o	o	o	o
Glutamine (17)	o	o	o	o	o	o
Branched-chain amino acids (16)	o	o	o	o	o	o
Whey protein (15)	o	o	o	o	o	o
MCTs (19)	o	o	o	o	o	o
Glycerol (20)	o	o	o	o	o	o
Ketones (18)	o	o	o	o	o	o
HMB (8)	o	o	o	o	o	o

Blood doping (9)	○	○	○	○	○	○
Anabolic steroids (10)	○	○	○	○	○	○
Androstenedione (11)	○	○	○	○	○	○
DHEA (12)	○	○	○	○	○	○
Stimulants (13)	○	○	○	○	○	○
Pyruvate (21)	○	○	○	○	○	○
Altitude training (22)	○	○	○	○	○	○
Ephedrine (23)	○	○	○	○	○	○
Other ergogenic aids not included in this list (24)	○	○	○	○	○	○

End of Block: Evaluation of Course Content

Start of Block: Interview Opportunity

Q41 Thank you for completing this survey. We really value your input and would appreciate your willingness to do a follow up interview that would last 15-20 minutes. Please provide your contact information below.

____

End of Block: Interview Opportunity
