# Supplementary material for: Educational Preparation and Course Approach of Undergraduate Sports Nutrition instructors in Large U.S. Institutions
Source: Sports (Basel). 2023 Sep 5;11(9):176. doi: 10.3390/sports11090176 (PMC10536551; doi:10.3390/sports11090176)
Supplement: Supplementary file 1 [file sports-11-00176-s001.zip › sports-2564794-supplementary tables.pdf]

Supplemental Table S1: Comparison of the average time spent, depth and importance of the major topics for **fats** reported by sports nutrition instructors in a given semester based on the instructor's degree, age and experience teaching the course. (N=41)

	Instructor's educational experience			Number of times course taught		
**Topic**	Both Nutrition and Exercise Physiology	Exercise Physiology Only	Nutrition Only	Novice (<3)	Experienced (3-6)	Veteran (>6)
**Structures**						
Time spent	6-10 min	6-10 min	6-10 min	6-10 min	6-10 min	6-10 min
Depth†	Moderate	Moderate	Moderate	Moderate	Moderate	Moderate
Importance‡	Moderate	Moderate	Moderate	Moderate	Moderate	Moderate
**Functions**						
Time spent	11-15 min	11-15 min	11-15 min	16-20 min	11-15 min	11-15 min
Depth	Moderate	Moderate	Moderate	Most	Moderate	Moderate
Importance	Moderate	Moderate	Most	Most	Moderate	Moderate
**Digestion and Absorption**						
Time spent	11-15 min	11-15 min	11-15 min	11-15 min	11-15 min	11-15 min
Depth	Moderate	Moderate	Moderate	Most	Moderate	Moderate
Importance	Moderate	Most	Moderate	Most	Moderate	Moderate
**Metabolic Pathways**						
Time spent	11-15 min	16-20 min	16-20 min	>20 min	16-20 min	11-15 min
Depth	Moderate	Most	Most	Most	Most	Moderate
Importance	Moderate	Most	Most	Most	Most	Most
**Utilization**						
Time spent	16-20 min	16-20 min	16-20 min	>20 min	16-20 min	16-20 min
Depth	Moderate	Most	Most	Most[a]	Most[b]	Most[b]
Importance	Moderate	Most	Most	Most	Most	Most
**Storage**						
Time spent	6-10 min	11-15 min	11-15 min	11-15 min	6-10 min	11-15 min
Depth	Moderate	Moderate	Most	Most	Most	Moderate
Importance	Moderate	Moderate	Most	Most	Most	Moderate
**Estimating needs**						
Time spent	16-20 min	11-15 min	11-15 min	16-20 min	16-20 min	11-15 min
Depth	Most	Moderate	Most	Moderate	Most	Most
Importance	Most	Moderate	Most	Moderate	Most	Moderate
**Dietary sources**						
Time spent	11-15 min	6-10 min	11-15 min	11-15 min	11-15 min	11-15 min
Depth	Moderate	Moderate	Moderate	Moderate	Moderate	Moderate
Importance	Moderate	Moderate	Most	Moderate	Moderate	Moderate
**Timing of recommendations**						
Time spent	16-20 min	6-10 min	11-15 min	16-20 min	11-15 min	11-15 min
Depth	Most	Moderate	Most	Moderate	Most	Moderate
Importance	Most	Moderate	Most	Most	Most	Moderate
**Fat loading**						
Time spent	6-10 min	6-10 min	6-10 min	11-15 min	6-10 min	6-10 min
Depth	Moderate	Moderate	Moderate	Moderate	Moderate	Moderate
Importance	Moderate	Moderate	Moderate	Moderate	Moderate	Moderate

†Categories of Depth scores based on a scale of 1-10

Least: 1-3.0

Moderate: 3.1-6.9

Most: 7.0 -10

‡Categories of Importance scores based on a scale of 1-10

Least: 1-3.0

Moderate: 3.1-6.9

Most: 7.0 -10

[a,b]Means within categories (degree type, age, experience) on each row are significantly different from each other; $P<0.001$

Supplemental Table S2: Comparison of the average time spent, depth and importance of the major topics for **body composition and weight management** reported by sports nutrition instructors in a given semester based on the instructor's degree, age and experience teaching the course. (N=41)

	Instructor's educational background				Number of times course taught		
**Topic**	Both Nutrition and Exercise Physiology	Exercise Physiology Only	Nutrition Only	< 35	Novice (<3)	Experienced (3-6)	Veteran (>6)
**Determination of healthy body weights**							
Time spent	11-15 min	11-15 min	11-15 min	11-15 min	6-10 min	6-10 min	11-15 min
Depth†	Moderate	Moderate	Most	Moderate	Most	Moderate	Moderate
Importance‡	Moderate	Moderate	Moderate	Most	Moderate	Moderate	Moderate
**Appropriate weight loss/gain strategies**							
Time spent	16-20 min	11-15 min	16-20 min	16-20 min	16-20 min	16-20 min	16-20 min
Depth	Moderate	Moderate	Most	Most	Most	Most	Most
Importance	Moderate	Moderate	Most	Most	Moderate	Most	Most
**Percent Body fat in males vs females**							
Time spent	6-10 min	6-10 min	6-10 min	6-10 min	6-10 min	6-10 min	6-10 min
Depth	Moderate	Moderate	Moderate	Moderate	Moderate	Moderate	Moderate
Importance	Moderate	Moderate	Moderate	Moderate	Moderate	Moderate	Moderate
**Sport specific percent body fat**							
Time spent	6-10 min	6-10 min	6-10 min	6-10 min	6-10 min	6-10 min	6-10 min
Depth	Moderate	Moderate	Moderate	Moderate	Moderate	Moderate	Moderate
Importance	Moderate	Moderate	Moderate	Moderate	Moderate	Moderate	Moderate
**General diet principles**							
Time spent	11-15 min	11-15 min	11-15 min	11-15 min	11-15 min	11-15min	11-15 min
Depth	Moderate	Moderate	Moderate	Most	Moderate	Moderate	Moderate
Importance	Moderate	Most	Most	Most	Moderate	Most	Most
**Prescribed diets**							
Time spent	6-10 min	6-10 min	11-15 min	11-15 min	1-5 min	11-15 min	6-10 min
Depth	Moderate	Moderate	Moderate	Most	Moderate	Moderate	Moderate
Importance	Moderate	Moderate	Moderate	Moderate	Moderate	Most	Moderate
**Body composition goals for athletes**							
Time spent	11-15 min	11-15 min	11-15 min	11-15 min	11-15 min	11-15 min	11-15min
Depth	Moderate	Moderate	Moderate	Most	Most[a]	Moderate[b]	Moderate[b]
Importance	Moderate	Most	Most	Most	Most	Most	Moderate
**Methods of determining**							

**body composition**							
Time spent	16-20 min	6-10 min	16-20 min	16-20 min	11-15 min	16-20 min	11-15min
Depth	Most	Moderate	Most	Most	Most	Most	Moderate
Importance	Most	Most	Most	Most	Most	Most	Most
**Methods of determining healthy body weight**							
Time spent	16-20 min	11-15 min	16-20 min	16-20 min	11-15 min	16-20 min	16-20 min
Depth	Most	Moderate	Most	Most	Most	Most	Most
Importance	Most	Moderate	Most	Most	Most	Most	Most
**Determining Energy Needs**							
Time spent	16-20 min	16-20 min	>20 min	>20 min	>20 min	16-20 min	16-20 min
Depth	Most	Most	Most	Most	Most	Most	Most
Importance	Most	Most	Most	Most	Most	Most	Most
**Changes in Body composition due to changes in life situations**							
Time spent	11-15 min	6-10 min	11-15 min	11-15 min	11-15 min	6-10 min	16-20 min
Depth	Moderate	Moderate	Moderate	Moderate	Moderate	Moderate	Moderate
Importance	Moderate	Moderate	Moderate	Moderate	Moderate	Moderate	Moderate
**Weight cutting practices**							
Time spent	11-15 min	6-10 min	11-15 min	11-15 min	6-10 min	11-15 min	11-15 min
Depth	Moderate	Moderate	Moderate	Moderate	Moderate	Moderate	Moderate
Importance	Moderate	Moderate	Moderate	Moderate	Moderate	Most	Moderate

†Categories of Depth scores based on a scale of 1-10

Least: 1-3.0

Moderate: 3.1-6.9

Most: 7.0 -10

‡Categories of Importance scores based on a scale of 1-10

Least: 1-3.0

Moderate: 3.1-6.9

Most: 7.0 -10

[a,b]Means within categories (degree type, age, experience) on each row are significantly different from each other; $P<0.001$

Supplemental Table S3: Comparison of time spent on the major topics for **vitamins and minerals** reported by sports nutrition instructors in a given semester based on the instructor's degree, age and experience teaching the course. (N=41)

	Instructor's educational background				Number of times course taught		
**Topic**	Both Nutrition and Exercise Physiology	Exercise Physiology Only	Nutrition Only	< 35	Novice (<3)	Experienced (3-6)	Veteran (>6)
**Vitamin A**							
Time spent	1-5 min	6-10 min	6-10 min	6-10 min	6-10 min	6-10 min	6-10 min
**Vitamin D**							
Time spent	11-15 min	6-10 min	11-15 min	6-10 min	11-15 min	6-10 min	11-15 min
**Vitamin E**							
Time spent	6-10 min	6-10 min	6-10 min	6-10 min	6-10 min	6-10 min	6-10 min
**Vitamin K**							
Time spent	1-5 min	1-5 min	6-10 min	6-10 min	6-10 min	1-5 min	6-10 min
**Vitamin B12**							
Time spent	6-10 min	6-10 min	6-10 min	6-10 min	6-10 min	6-10 min	6-10 min
**Folic Acid**							
Time spent	6-10 min	6-10 min	6-10 min	6-10 min	6-10 min	6-10 min	6-10 min
**Other B Vitamins**							
Time spent	6-10 min	6-10 min	6-10 min	6-10 min	6-10 min	6-10 min	6-10 min
**Vitamin C**							
Time spent	6-10 min	6-10 min	6-10 min	6-10 min	6-10 min	6-10 min	6-10 min
**Calcium**							
Time spent	11-15 min	6-10 min	11-15 min	6-10 min	11-15 min	6-10 min	6-10 min
**Sodium**							
Time spent	16-20 min	6-10 min	11-15 min	11-15 min	11-15 min	11-15 min	11-15 min
**Potassium**							
Time spent	11-15 min	6-10 min	11-15 min	11-15 min	11-15 min	11-15 min	6-10 min
**Iron**							
Time spent	16-20 min	6-10 min	11-15 min	11-15 min	11-15 min	11-15 min	11-15 min
**Other Minerals**							
Time spent	1-5 min	6-10 min	6-10 min	1-5 min	6-10 min	1-5 min	6-10 min

No significant difference between groups

Supplemental Table S4: Comparison of time spent on the major topics for **ergogenic aids** reported by sports nutrition instructors in a given semester based on the instructor's degree, age and experience teaching the course. (N=41)

	**Instructor's educational background**			**Number of times course taught**		
**Topic**	Both Nutrition and Exercise Physiology	Exercise Physiology Only	Nutrition Only	Novice (<3)	Experienced (3-6)	Veteran (>6)
**Caffeine**						
Time spent	11-15 min	11-15 min	11-15 min	11-15 min	11-15 min	11-15 min
**Sodium Bicarb**						
Time spent	1-5 min	6-10 min	6-10 min	6-10 min	6-10 min	6-10 min
**Creatine**						
Time spent	11-15 min	11-15 min	11-15 min	16-20 min	11-15 min	11-15 min
**Nitrate**						
Time spent	6-10 min	6-10 min	6-10 min	6-10 min	6-10 min	6-10 min
**Beta-alanine**						
Time spent	1-5 min	6-10 min	6-10 min	6-10 min	6-10 min	6-10 min
**Leucine**						
Time spent	11-15 min	11-15 min	11-15 min	11-15 min	11-15 min	11-15 min
**Carnitine**						
Time spent	1-5 min	6-10 min	6-10 min	6-10 min	1-5 min	6-10 min
**Glutamine**						
Time spent	6-10 min	6-10 min	6-10 min	6-10 min	6-10 min	6-10 min
**BCAA's***						
Time spent	11-15 min	11-15 min	11-15 min	11-15 min	11-15 min	11-15 min
**Whey protein**						
Time spent	11-15 min	11-15 min	11-15 min	16-20 min	11-15 min	11-15 min
**MCTs**						
Time spent	6-10 min	6-10 min	6-10 min	11-15 min	6-10 min	6-10 min
**Glycerol**						
Time spent	1-5 min	1-5 min	1-5 min	1-5 min	1-5 min	1-5 min
**Ketones**						
Time spent	6-10 min	6-10 min	6-10 min	6-10 min	6-10 min	6-10 min
**HMB**						
Time spent	1-5 min	6-10 min	1-5 min	1-5 min	6-10 min	1-5 min
**Blood doping**						
Time spent	1-5 min	1-5 min	1-5 min	1-5 min	6-10 min	1-5 min
**Anabolic steroids**						
Time spent	1-5 min	6-10 min	6-10 min	1-5 min	6-10 min	1-5 min
**Androstenedione**						
Time spent	1-5 min	1-5 min	1-5 min	1-5 min	6-10 min	1-5 min
**DHEA**						
Time spent	1-5 min	1-5 min	1-5 min	1-5 min	6-10 min	1-5 min
**Stimulants**						
Time spent	6-10 min	6-10 min	6-10 min	6-10 min	6-10 min	6-10 min
**Pyruvate**						
Time spent	1-5 min	1-5 min	1-5 min	1-5 min	1-5 min	1-5 min
**Altitude training**						
Time spent	1-5 min	1-5 min	6-10 min	6-10 min	1-5 min	6-10 min

**Ephedrine**						
Time spent	1-5 min	1-5 min	1-5 min	1-5 min	6-10 min	1-5 min
**Other ergogenic aids** Time spent	6-10 min	6-10 min	6-10 min	6-10 min	6-10 min	6-10 min

* Branched chain amino acids

No significant differences between groups
